# Conserved and Taxon-Specific Patterns of Phenotypic Modularity in the Mammalian Dentition

**DOI:** 10.1093/iob/obac017

**Published:** 2022-04-28

**Authors:** Risa Takenaka, Selene M Clay, Sunwoo Yoo, Leslea J Hlusko

**Affiliations:** Department of Integrative Biology, University of California, Berkeley, Berkeley, CA 94720, USA; Department of Integrative Biology, University of California, Berkeley, Berkeley, CA 94720, USA; Department of Integrative Biology, University of California, Berkeley, Berkeley, CA 94720, USA; Department of Integrative Biology, University of California, Berkeley, Berkeley, CA 94720, USA; Museum of Vertebrate Zoology, University of California, Berkeley, Berkeley, CA 94720, USA; Human Evolution Research Center, University of California, Berkeley, Berkeley, CA 94720, USA

## Abstract

Previous genotype:phenotype mapping of the mouse and primate dentition revealed the presence of pre- and post-canine modules in mice and anthropoid primates, as well as molar and premolar submodules in anthropoid primates. We estimated phenotypic correlation matrices for species that sample broadly across Mammalia to test the hypothesis that these modules exist across a broader range of taxa and thereby represent a conserved mammalian trait. We calculated phenotypic correlation matrices from linear dental measurements of 419 individual specimens representing 5 species from 4 mammalian orders: Artiodactyla (*Odocoileus hemionus*), Carnivora (*Canis latrans* and *Ursus americanus*), Didelphimorphia (*Didelphis virginiana*), and Primates (*Colobus guereza*). Our results based on hierarchical clustering indicate a generally higher correlation within incisors and among post-canine teeth. However, the post-canine phenotypic correlation matrices do not consistently exhibit the premolar and molar submodularity observed in anthropoid primates. Additionally, we find evidence of sex differences in the *Odocoileus* phenotypic correlation matrices: Males of this species exhibit overall higher inter-trait correlations compared to females. Our overall findings support the interpretation that incisors and post-canine dentition represent different phenotypic modules, and that this architecture may be a conserved trait for mammals.

## Spanish

Un mapeo previo del genotipo:fenotipo de la dentición de ratones y primates reveló la presencia de módulos precaninos y poscaninos en ratones y primates antropoides, además de molares y premolares en primates antropoides. Estimamos matrices de correlación fenotípica para especies que representan una muestra amplia de Mammalia, para probar la hipótesis de que estos módulos existen en una gama más amplia de taxones y, por lo tanto, representan un rasgo conservado de mamíferos.

Calculamos matrices de correlación fenotípica a partir de medidas dentales lineales de 419 especímenes individuales que representan cinco especies de cuatro órdenes de mamíferos: Artiodactyla (*Odocoileus hemionus*), Carnivora (*Canis latrans* y *Ursus americanus*), Didelphimorphia (*Didelphis virginiana*) y Primates (*Colobus guereza*). Los resultados, basados en el agrupamiento jerárquico, indican que hay una correlación generalmente más alta entre los incisivos y entre los dientes poscaninos. Sin embargo, las matrices de correlación fenotípica de los poscaninos no exhiben consistentemente la submodularidad del premolar y molar observada en primates antropoides. Adicionalmente, encontramos evidencia de dimorfismo sexual en las matrices de correlación fenotípica de *Odocoileus*: los machos de esta especie exhiben, en general, correlaciones entre rasgos más altos que las hembras. Nuestro descubrimiento general apoya la interpretación de que los incisivos y la dentición poscanina representan diferentes módulos fenotípicos, y que esta arquitectura puede ser un rasgo conservado para los mamíferos.

## Introduction

Heterodonty is a key feature of mammalian evolution that facilitated the clade's radiation and diversification (Bergqvist 2003; Luo 2007; Price et al. 2012). The typical heterodont mammalian dentition consists of four tooth types: incisors, canines, premolars, and molars (Hillson 2005). Both extinct and extant mammalian groups exhibit variation on this basic form resulting from phylogenetic history, ecological adaptation, and developmental and functional constraints (e.g., Janis and Fortelius 1988; Evans and Sanson 2003; Evans et al. 2007; Goswami et al. 2011; Forasiepi and Sánchez-Villagra 2014). Fundamental to the evolutionary history of the mammalian dentition is the relationship between genotype and phenotype.

Cheverud (1988) proposed that phenotypic correlations could serve as proxies for genetic correlations, and consequently, provide insight into the relationship between genotype and phenotype. Many studies have bolstered this interpretation, especially for traits that are highly heritable (Roff 1995; Cheverud 1996a; Marroig and Cheverud 2005; Hlusko et al. 2011; Sodini et al. 2018; Hardin 2020; Paul et al. 2020). Variation in tooth size has a large genetic contribution, making it a particularly useful phenotypic proxy for the underlying genetic architecture (Dempsey and Townsend 2001; Rizk et al. 2008; Hlusko et al. 2011; Hughes et al. 2000).

Close observation of the patterns of variation within and between anatomical structures often reveals patterns of nested correlation, where variation is highly correlated within a suite of traits and uncorrelated with variation in other sets of traits. This concept is known as phenotypic integration (Olson and Miller 1958). Patterns of anatomical variation are thought to represent the modular influences of genetics, development, and/or functional mechanisms that influence the evolutionary response of the phenotype (e.g., Cheverud 1996b; Pigliucci and Preston 2004; Schlosser and Wagner 2004; Klingenberg 2008; Esteve-Altava 2017a). For the purposes of this study, we define a module as a set of linear measurements that form a subset of an anatomical structure within which variation is highly correlated relative to other linear measurements of that structure. We define submodules as modules that are nested within a larger module. These definitions align with the common use of the term morphological modularity, which is also known as variational modularity (Klingenberg 2014).

**Fig. 1:**
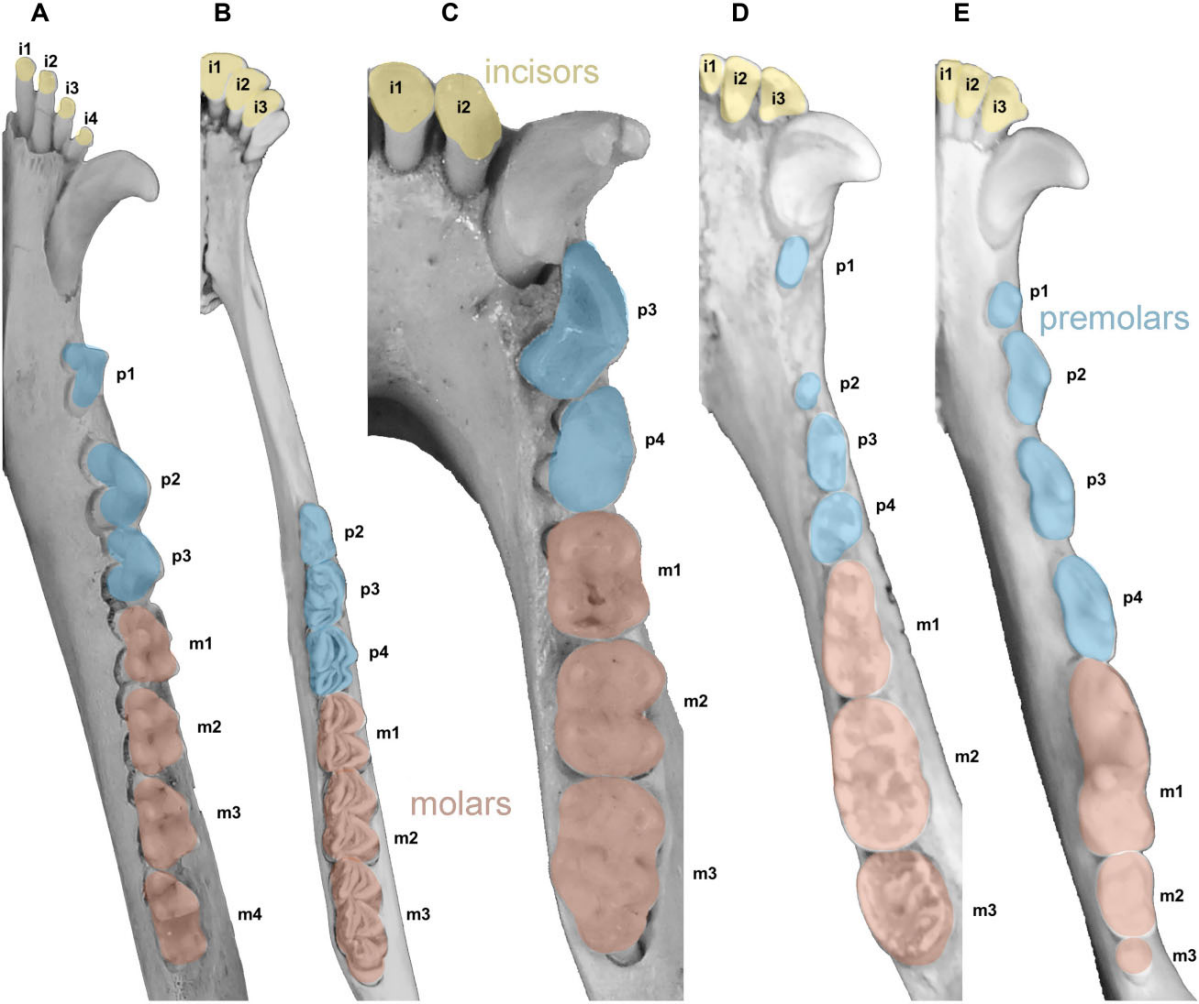
Representative mandibular dentitions of the five taxa included in this study. (A) *D. virginiana*, (B) *O. hemionus*, (C) *C. guereza*, (D) *U. americanus*, and (E) *C. latrans*. All are shown in occlusal view with the labial surface oriented to the top of the figure (not to scale). Each tooth is annotated according to Hillson (2005).

Dental variation is an ideal system through which to apply a morphological modular approach. First, variation in tooth size is highly heritable (Hughes et al. 2000; Dempsey and Townsend 2001; Rizk et al. 2008; Hlusko et al. 2011). Second, the dentition is an anatomical structure with subsets. The typical mammalian dentition is heterodont and consists of four tooth types: incisors in the anterior region of the dental arcade, followed by a canine, a set of premolars, and finally, a set of molars (see Fig. 1). Previous studies have suggested patterns of pleiotropic effects between and within these tooth classes. For example, developmental-genetic investigations of the mouse dentition provide evidence of developmental modularity that corresponds, to some degree, to tooth types (reviewed in Yu and Klein 2020). Quantitative genetic research has also provided evidence of genetic modularity corresponding to tooth types (Hlusko et al. 2011; Hardin 2020). These results imply that separate, though hypothetical, gene expression territories may be responsible for variation between and within tooth classes in primate and rodent species (Hlusko et al. 2006, 2011; Hlusko and Mahaney 2007, 2009; Grieco et al. 2013). The findings from these studies are corroborated by the deep evolutionary history of the fossil record that further demonstrates the fundamental nature of these tooth types (Ungar 2010). Together, these lines of evidence provide justification for pursuing a morphological variational approach to gain insight into the genetic architecture underlying the mammalian heterodont dentition.

We base our study on patterns of genetic correlations estimated through quantitative genetic analyses. As mentioned previously, variation in tooth size is highly heritable, with heritability (*h*^2^) being the estimation of the proportion of the phenotypic variance that can be attributed to additive genetic variance (Rizk et al. 2008). As phenotypic variation can be decomposed into a genetic and a non-genetic component, phenotypic correlations can also be decomposed into genetic and non-genetic components. The genetic correlation or covariance between two phenotypic measurements can be estimated for populations with known pedigree structures (e.g., Hlusko et al. 2006). A matrix summarizing these additive genetic variances and covariances, known as the *G*-matrix, is the foundation for interpretations of genetic modularity (reviewed in Lynch and Walsh 1998; Steppan et al. 2002; McGuigan 2006; Arnold et al. 2008; but see Pigliucci 2006 for critique on this approach).

Hlusko and colleagues (Hlusko and Mahaney 2009; Hlusko et al. 2011) calculated genetic correlation matrices of mouse and baboon dentition and detected two genetic modules: an incisor module and a separate module that consists of the post-canine teeth (i.e., molars and premolars). Quantitative genetic analyses of macaque dentitions similarly demonstrate anterior and post-canine genetic modules (Hardin 2020). There is also evidence of genetic submodularity between the premolars and molars within the post-canine dentition of baboons (Hlusko and Mahaney 2009; Hlusko et al. 2011) and phenotypic evidence across a sample of cercopithecids (Grieco et al. 2013). While quantitative genetic analyses of dental variation in brown-mantled tamarins (Hardin 2019) do not reveal evidence of genetic modularity, it is possible that the dramatic body size reduction in tamarins may have resulted in a derived dental genetic architecture.

Phenotypic modularity has been used to infer genetic modularity, an assumption defined through an analysis of morphological variation and deeply embedded in the study of variational modularity (Esteve-Altava 2017b; Mitteroecker 2009). This assumption has been challenged, however, as pleiotropic factors can replicate a modular covariance structure at the phenotypic level, and therefore, variational modules are not necessarily evidence of genetic modularity (Mitteroecker 2009). With this caveat in mind, we utilize morphological modularity, or variational modularity, to test hypotheses about genetic modularity in the heterodont mammalian dentition.

In this study, we use phenotypic data to investigate the idea that the anterior and post-canine dental genetic modules in primates and mice represent an ancestral condition for eutherians, and perhaps therians more broadly. We use linear dental measurements from four eutherian genera (Artiodactyla [*Odocoileus*], Carnivora [*Canis*, *Ursus*], Primates [*Colobus*]) and a marsupial (Didelphimorphia [*Didelphis*]) to calculate phenotypic correlation matrices to test two specific hypotheses:

1. The pre- and post-canine modules observed in baboons and mice characterize the modularity of other mammalian taxa.
2. The submodularity observed for the baboon post-canine module characterizes a broader sampling of mammalian taxa.

## Materials and methods

### Materials

**Fig. 2:**
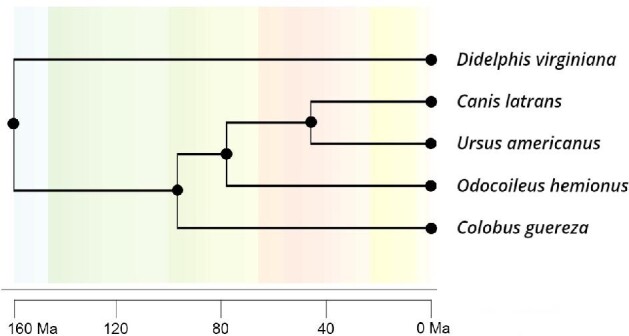
Molecular phylogeny of the five taxa included in this study, estimated using *Timetree* (Kumar et al., 2017).

Data were collected from the teeth of skeletonized maxillae and mandibles from 419 crania housed in the following museum collections: American Museum of Natural History (New York, NY, USA); Cleveland Museum of Natural History (Cleveland, Ohio, USA); University of California's Museum of Vertebrate Zoology (Berkeley, CA, USA); and Smithsonian Institution's National Museum of Natural History (Washington, D.C., USA). Our sample consists of one species of Artiodactyla (*Odocoileus hemionus hemionus*), two Carnivora (*Canis latrans lestes* and *Ursus americanus*), one Didelphimorphia (*Didelphis virginiana virginiana*), and one Primate (*Colobus guereza*) (see Table 1 for species-specific sample sizes). Taxonomic identification follows the convention used by each of the museum collections. These taxa were chosen because they reflect variation in the mammalian dentition (Fig. 1, Supplementary Fig. 1, Table 2) and because they had large enough sample sizes to confidently estimate phenotypic correlation matrices. The estimated molecular divergence times based on Timetree (Kumar et al. 2017) are shown in Fig. 2.

**Table 1:** Sample sizes of each species included in this analysis

	*C. latrans*	*O. hemionus*	*U. americanus*	*D. virginiana*	*C. guereza*
Females	40	39	14	40	49
Males	40	38	38	17	60
Unknown sex	0	0	28	0	16
Total	80	77	80	57	125

**Table 2:** Brief description of species included in this analysis

Order	Species	Dental formulaI, C, P, M	Diet	Average body weight
Artiodactyla	*O. hemionus*	0/3, 0/1, 3/3, 3/3	Herbivorous^1^	Male: 100 kg Female: 65 kg^6^
Carnivora	*C. latrans*	3/3, 1/1, 4/4, 2/3	Primarily carnivorous, but opportunistic omnivore²	Male: 11.1 kg Female: 9.9 kg²
Carnivora	*U. americanus*	3/3, 1/1, 4/4, 2/3	Omnivorous diet centered on vegetation³	Male: 86 kg Female: 58 kg³
Didelphimorphia	*D. virginiana*	5/4, 1/1, 3/3, 4/4	Omnivorous diet of insects and carrion, as well as fruits and grains⁴	Male: 2.8 kg Female: 1.9 kg⁴
Primate	*C. guereza*	2/2, 1/1, 2/2, 3/3	Primarily folivorous⁵	Male: 11.8 kg Female: 8.3 kg^6^

Dental formulae are from Hillson (2005). From left to right are the numbers of incisors (I), canines (C), premolars (P), and molars (M). Top and bottom of the fraction correspond to maxillary and mandibular teeth, respectively. For example, 0/3 for *O. hemionus* incisors indicates zero maxillary incisors and three mandibular incisors. Diet and body weight references: ^1^Anderson and Wallmo 1984, ²Bekoff 1977, ³Larivière 2001, ⁴McManus 1974, ⁵Harris 2006, and ^6^Grzimek 1990.

The ratio of the number of male to female samples is roughly equal for both *C. latrans* and *O. hemionus*, skews toward females for *D. virginiana*, and skews slightly male for *C. guereza* (Table 1). Roughly a third of *U. americanus* specimens were of unknown sex and the remaining two-thirds skew toward males. All of the species surveyed exhibit sex differences with respect to body size, with males being larger than the females (Table 2).

### Data collection

For each tooth, we collected the linear measurements of the maximum mesiodistal distance (i.e., the maximum length) and the maximum buccolingual or labiolingual distance (i.e., the maximum width; the term buccolingual refers to molar and premolars, and labiolingual to incisors and canines; see Hillson 2005). We collected these data from adult crania with fully erupted permanent dentition, and we excluded measurements from teeth that were absent (e.g., a common issue for *U. americanus* premolars, likely lost during specimen preparation), chipped, cracked, diseased, or excessively worn. These measurements were collected by hand using Mitutoyo calipers. Each specimen's dentition was measured three times from both the left and right sides of the maxillae and mandibles. Inter- and intra-observer measurement errors were calculated to measure precision, with the differences between observers and measurement rounds being 3.9 percentage of the average measurement. We used the average of the three measurements for all further analyses. We used *C. guereza* data that were previously collected for another study (see Grieco et al. 2012, 2013). All data used for the final analyses are available in Supplementary File 1.

### Analytical methods

Our analytical approach consists of three components. First, we estimated phenotypic correlation matrices for each taxon. Second, we used a hierarchical clustering approach to compare the number of modules in the dental phenotypic correlation matrices across all of the taxa. Third, we employed hypothesis testing using the Fisher Z-transformation to look for sex differences within each species. Details for each of these components are provided below.

Although we collected both mesiodistal and buccolingual/buccolabial measurements for each tooth, we focused our analyses on the mesiodistal measurements. We include results of analyses with both mesiodistal and buccolingual/buccolabial measurements in the supplementary figures. This decision is based on results from baboon quantitative genetic analyses that report a genetic correlation between buccolingual width and body size (Hlusko et al. 2006). Therefore, the incorporation of buccolingual tooth dimensions may complicate our analysis by potentially including the genetic effects of body size variation (see Hlusko et al. 2016, Monson et al. 2019 for further discussion).

#### Correlation matrices

Phenotypic correlation matrices for tooth size measurements were estimated for all possible pairwise comparisons within a taxon using Pearson's pairwise correlation, following Grieco et al. (2013). All statistical analyses were executed in R/3.4.1 (R Core Team, 2019). The corrplot package in R (v0.84, Wei and Simko 2017) was used to find correlations significant at the 5% significance level. For each correlation matrix, we also applied both a false discovery rate (FDR) of 0.05 and a Bonferroni correction to the *P*-values.

#### Modularity assessment

To identify phenotypic modules of tooth size variation, we performed hierarchical clustering using the corrplot package in R (v0.84, Wei and Simko 2017). Hierarchical clustering groups similar objects into clusters or modules to identify structures or patterns in a matrix (Friedman et al. 2001). We ran this test separately for the maxillary and mandibular datasets of each species. We also excluded the canines because they were not included in the baboon quantitative genetic analyses (Hlusko et al. 2011) and their modular structure is uncertain (Hardin 2019, 2020; Paul et al. 2021). Because three tooth classes are represented in the analyses, we set the method to identify between three and five specific clusters. Common statistical methods that examine modularity or matrices were considered (e.g., Klingenberg 2009; Klingenberg and Marugán-Lobón 2013; Adams 2016), but these methods were designed for analyzing spatial data and are therefore incompatible with our linear measurements.

#### Sex differences

To look for sex differences within each species, we performed multiple hypothesis tests comparing the differences between the male and female sample correlations between each tooth pair. We used the z-scores obtained after applying the Fisher z-transformation (Fisher 1915) to the sample correlations. We excluded the maxillary P2 and mandibular p3 from *U. americanus* for this analysis due to high missingness. To control for multiple testing, we applied a Bonferroni correction to the *P*-values from the hypothesis tests.

### Abbreviations

We refer to specific teeth using a combination of letters and numbers that correspond to tooth type and position. The abbreviations we use are i = incisor; c = canine; p = premolar; and m = molar. Capital letters refer to maxillary teeth, and lower-case letters refer to mandibular teeth. The number after the letter indicates the tooth position: For example, m2 refers to the second mandibular molar, and P2 refers to the second maxillary premolar.

## Results

### Descriptive univariate statistics

For each measurement, we calculated the number of samples collected, minimum value, first quartile, median, mean, third quartile, maximum value, and standard deviation, and tested to see if the values followed a normal distribution using the Shapiro–Wilk test (Supplementary Table 1). About 16% of the measurements deviate from a normal distribution (Supplementary Table 2). However, these non-normally distributed measurements were not consistently found in any one dental submodule and therefore are unlikely to bias our general assessment. We did not find evidence for significant differences between corresponding measurements on the right and left side of the dental arcade based on correlation matrices (data not shown). Therefore, we used the measurements from the right side for all subsequent analyses.

### Phenotypic correlation matrices: inter-taxon comparisons

**Fig. 3:**
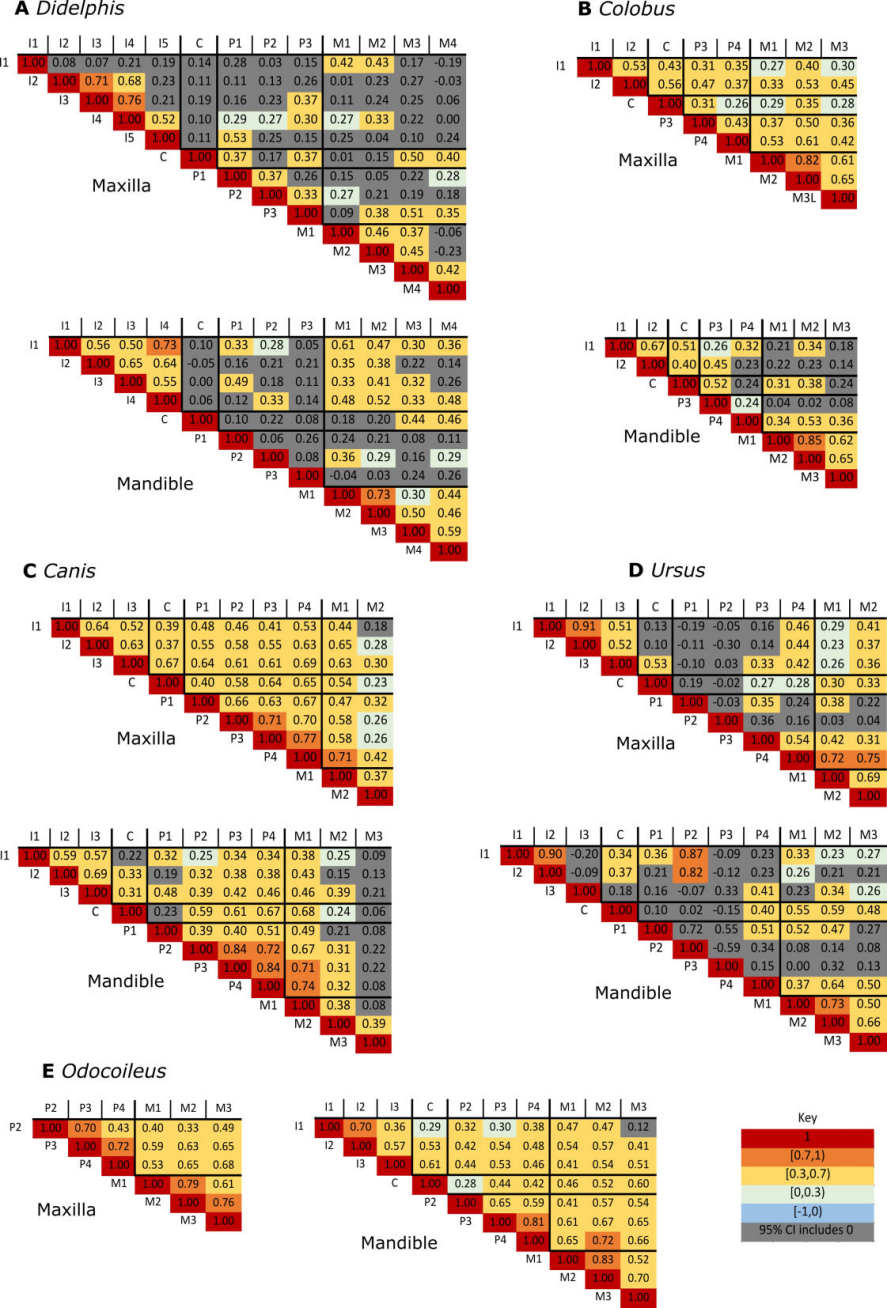
Phenotypic correlation matrices for tooth length. (A) *D. virginiana*, (B) *C. guereza*, (C) *C. latrans*, (D) *U. americanus*, and (E) *O. hemionus*. The strength of the correlation is denoted by the color, with higher correlations in red and reducing through orange to yellow, negative correlations in blue, and non-significant correlations in gray. Note that the diagonal correlations of 1.0 are reporting the correlation between a measurement and itself. Abbreviations: M: molar, P: premolar, C: canine, and I: incisor. Number corresponds to tooth position, such that M4 is the fourth molar. Only the teeth on the right side of the dental arcade are shown. These matrices are based on mesiodistal (length) measurements. The full matrix (with length and width) can be found in Supplementary Fig. 1.

Correlation matrices of all possible pairwise comparisons in both the mandibular and maxillary dentition for all five species are presented in Fig. 3 (length measurements only) and Supplementary Fig. 2 (length and width measurements). We calculated the mean of all length and width pairwise correlations (excluding the main diagonal, which have a correlation of 1.0) within and between each tooth class and for the overall matrix on the right side of the dentition (Supplementary Table 3).

The matrices in Fig. 3 are shown with uncorrected *P*-values. To account for multiple testing, we applied the FDR and Bonferroni corrections. Applying an FDR < 0.05 shows the same general patterns as the non-corrected matrices (Supplementary Fig. 3, Supplementary Fig. 4). When the more stringent Bonferroni correction is applied to our *P*-values, we still see the same fundamental patterns of correlations, although there are more non-significant results (Supplementary Fig. 3, Supplementary Fig. 4).

For this broad sampling of mammalian taxa, we do not always observe highest correlations within tooth classes, unlike the results previously reported in mice (Hlusko et al. 2011) and primates (Hlusko and Mahaney 2009; Hlusko et al. 2011; Grieco et al. 2013). However, we do find stronger correlations within the molar tooth class (i.e., a molar module) except for the *C. latrans* mandible (Table 3, Supplementary Fig. 2). Furthermore, we find stronger correlations among incisors (i.e., an incisor module) in the following dataset: *C. latrans* mandible, *C. guereza* maxilla, and to a lesser extent, in the *D. virginiana* mandible (Fig. 3, Supplementary Fig. 2, Supplementary Table 3). We only observe distinct post-canine modules (i.e., stronger correlations between molars and premolars compared to overall correlations) in *C. guereza* maxilla, and to a lesser extent, in the *U. americanus* maxilla (Supplementary Table 3; see Grieco et al. 2013 for *C. guereza*).

### Hierarchical clustering: species-specific patterns

To look for patterns of modularity, we examined the phenotypic correlation matrices using hierarchical clustering. We analyzed the maxilla and mandible for each taxon independently, focusing on mesiodistal-length measurements (see Section 2.3 for justification).

**Fig. 4:**
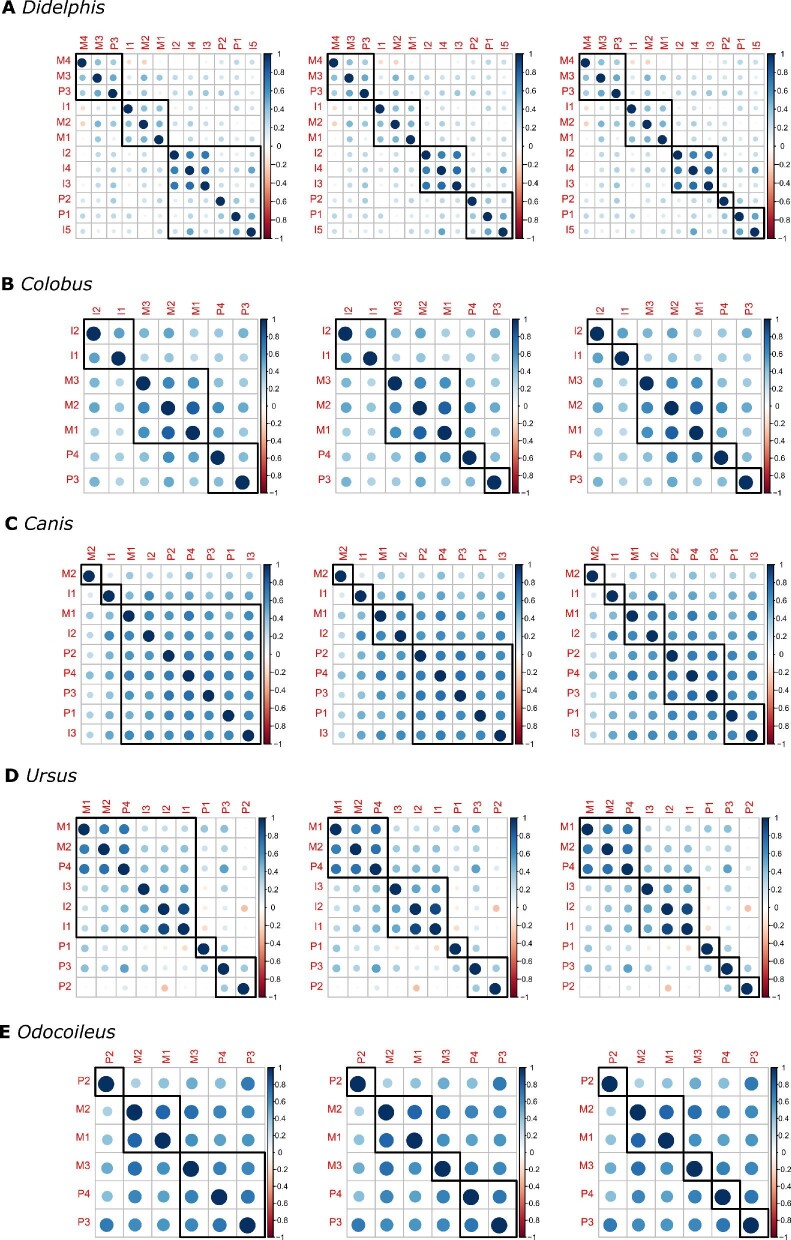
Hierarchical clustering results for the maxillary dentition. From left to right, panels show three, four, and five clusters for each taxon. The strength of the correlation is denoted by color and dot size, with stronger correlations in darker and larger dots. Blue and red dots indicate positive and negative correlation, respectively. Abbreviations: M: molar, P: premolar, C: canine, and I: incisor. Number corresponds to tooth position, such that M2 is the second molar.

**Fig. 5:**
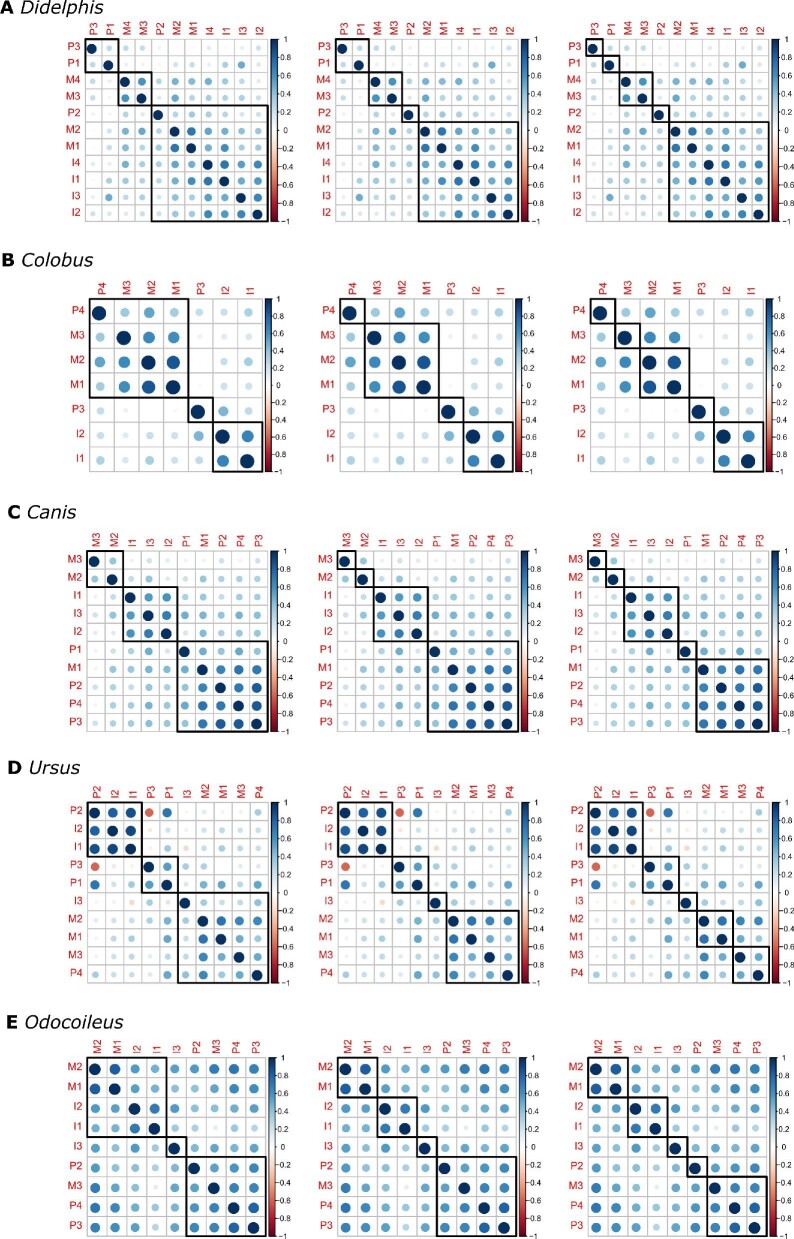
Hierarchical clustering results for the mandibular dentition. From left to right, panels show three, four, and five clusters for each taxon. The strength of the correlation is denoted by color and dot size, with stronger correlations in darker and larger dots. Blue and red dots indicate positive and negative correlation, respectively. Abbreviations: M: molar, P: premolar, C: canine, and I: incisor. Number corresponds to tooth position, such that M2 is the second molar.

In the *D. virginiana* maxilla, we find a module consisting of the three central incisors (I2, I3, I4). The most mesial and distal incisors (I1 and I5, respectively) are absent from this clustering (Fig. 4a). In the mandible, all four incisors cluster together in a module that also includes some post-canine dentition (Fig. 5a). The two most distal molars (m3 and m4) also consistently group together in the mandible (Fig. 5a). Overall patterns of correlations were generally weak in *D. virginiana*, but our results suggest the presence of an incisor module in both the maxilla and mandible.

We find distinct molar, premolar, and incisor modules in the *C. guereza* maxilla (Fig. 4b). This result is consistent with those previously described by Grieco and colleagues (2013). In the mandible, we find a suggestive molar module as well as a distinct incisor module (Fig. 5b). However, unlike in the maxilla, the two mandibular premolars do not cluster together. The phenotypic independence between the mandibular p3 and p4 in *C. guereza* in our sample is not surprising, given that the p3 of most anthropoid primates, including cercopithecoids, is derived and forms a honing complex with the maxillary canine (Swindler 2002). Our results indicate that both the maxilla and mandible of *C. guereza* have distinct and separate incisor and post-canine modules.

We observe interesting patterns involving the carnassial teeth (maxillary P4 and mandibular m1) in *C. latrans.* In the maxilla, correlations are centered around the carnassial (P4) and dissipate away from it (Fig. 4c). In the mandible, we see that the carnassial (i.e., the first molar) groups with premolars (Fig. 5c). Additionally in the mandible, we find that the incisors separate from the post-canine dentition and form their own module (Fig. 5c).

There is a suggestive incisor module, premolar module, and molar module in the *U. americanus* maxilla (Fig. 4d). We also find that the molars cluster together in the mandible, as do the two central incisors (Fig. 5d). The third, most distal incisor separates from the two central incisors in the mandible (Fig. 5d). Interestingly, we find that the fourth premolar, which is the premolar located next to the first molar, clusters with molars instead of with the other premolars in both the maxilla and mandible (Fig. 5d).

Finally, we see strong correlations among all teeth in *O. hemionus*. In the maxilla, which lacks both the canine and incisors, all molars and premolars are highly correlated with no clear modules separating the two tooth classes (Fig. 4e). There are also strong correlations across the mandible, with suggestive incisor, molar, and premolar clusters (Fig. 5e). However, the most distal molar (m3) groups with the premolars instead of with the other molars (Fig. 5e). Similar to the pattern observed in the *U. americanus* mandible, the most distal incisor (i3) is separated from the two central incisors (Fig. 5e).

Overall, the hierarchical clustering results suggest a distinct incisor module that is separate from the post-canine dentition. We observe this incisor module in the mandible of all species surveyed in this study. We find incisor modules in the maxilla with the exception of *C. latrans* and *O. hemionus* (the latter of which lack maxillary incisors entirely).

### Sex differences

We examined the difference between the individual correlations from each pair of teeth between male and female individuals using hypothesis testing with the Fisher z-transformation. For this analysis, we used mesiodistal-length measurements from the right side of the dental arcade. None of the comparisons were significant at a Bonferroni threshold; however, 44% of measurements in the *O. hemionus* mandible had a raw *P*-value < 0.05, which was the highest proportion of all species (Supplementary Table 4).

**Fig. 6:**
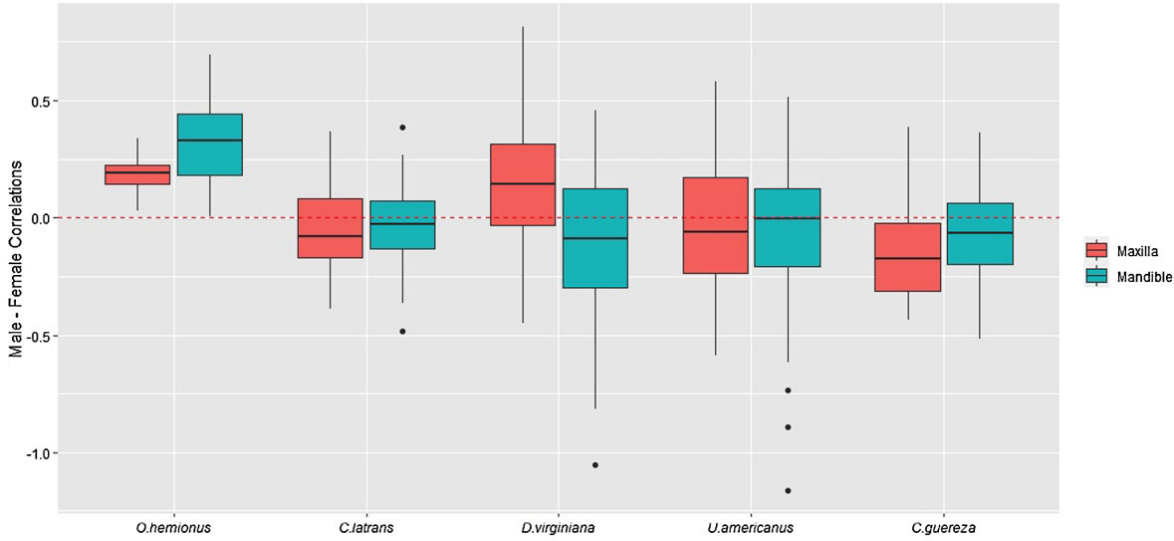
Distribution of the differences between male and female correlations. Each box plot represents the female correlations for each tooth pair subtracted from the corresponding male correlations. The horizontal red line at 0 indicates the value at which there is no difference between male and female correlations. Results for maxilla are in red, and mandible in blue.

We then looked at the distribution of the differences between male and female correlations (Fig. 6, Supplementary Fig. 5). We did not see any differences between males and females for any of the taxa except for *O. hemionus*, where we observe that males have higher correlations compared to females for corresponding tooth pairs.

**Fig. 7:**
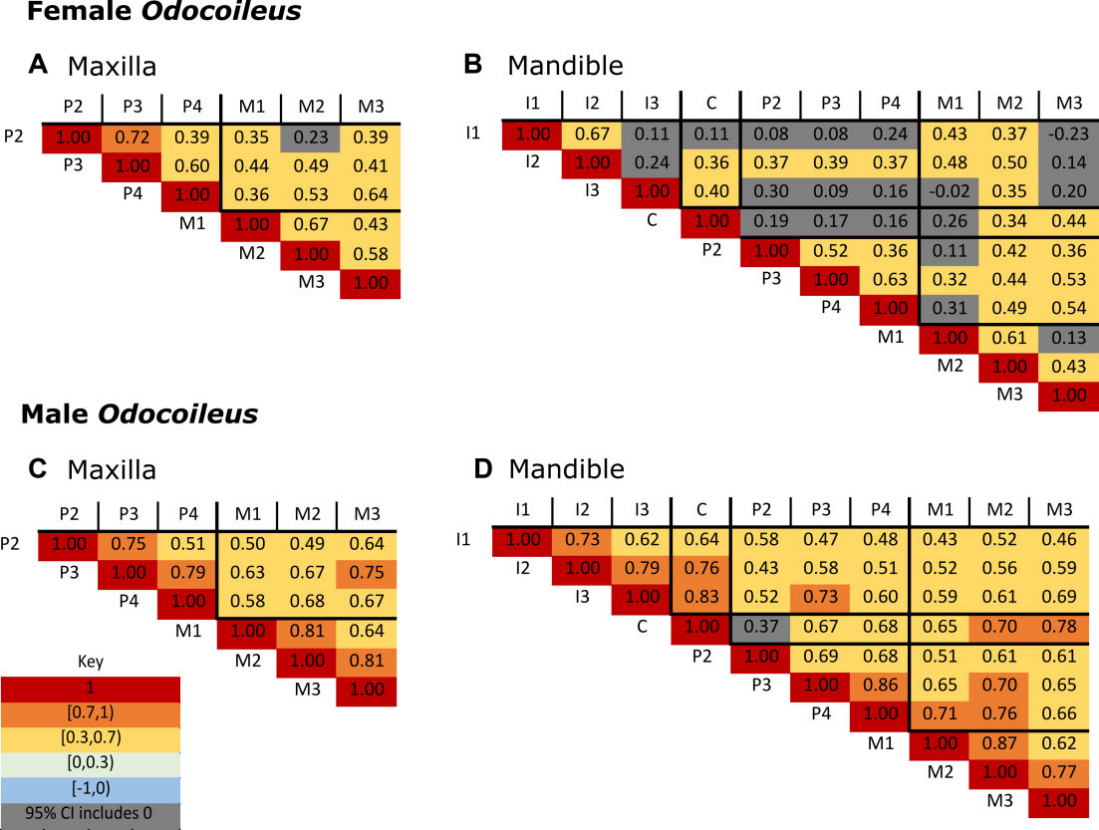
Sex-specific *O. hemionus* correlation matrices for tooth length. (A) female maxilla, (B) female mandible, (C) male maxilla, (D) male mandible. Note that Odocoileus lack incisors and canines in their maxillary dentition. Additionally, the diagonal correlations of 1.0 are reporting the correlation between a measurement and itself. Abbreviations: M: molar, P: premolar, C: canine, and I: incisor. Number corresponds to tooth position, such that M2 is the second molar.

We also see a qualitative difference between the male and female phenotypic correlation matrices of *O. hemionus* in both the mandible and maxilla, the male matrices have stronger and more significant correlations compared to female matrices (Fig. 7, Supplementary Fig. 2). Furthermore, when we calculated the mean pairwise correlations for female and male maxillae independently, the female pairwise trait comparisons yielded overall less-significant correlations compared to the male dataset (mean = 0.41 and 0.54 for female and male, respectively; Supplementary Table 3). This sex difference in overall pairwise trait comparison is even more pronounced in the mandible (mean = 0.28 and 0.54 for female and male, respectively; Supplementary Table 3). Of the taxa included in our study, this pattern of sex difference was unique to *O. hemionus*.

## Discussion

We estimated phenotypic correlation matrices of dental linear metrics for five genera, sampled from four mammalian orders, to test two hypotheses: (1) That the pre- and post-canine modules observed in baboons and mice characterize the modularity of other mammalian taxa; and, (2) that the submodularity observed for the baboon post-canine module characterizes a broader sampling of mammalian taxa. Our results provide suggestive evidence in support of the first hypothesis and reject the second. Additionally, hypothesis testing demonstrates that there is no significant difference between the individual correlations from each pair of teeth for males and females of the same species. However, we observe a qualitative difference in the intensity of correlation between the male and female correlation matrices of *Odocoileus*, the mule deer. We discuss each of these results in detail below.

Many taxa included in this study exhibit correlations within incisors and within the post-canine teeth that were higher than correlations among incisors and post-canine teeth. Our hierarchical clustering analysis enabled us to partition the phenotypic correlation matrices into three, four, and five clusters. For all taxa in our study, the incisors are generally in the same cluster, no matter the number of clusters created. This fairly constant pattern of incisor-clustering is what would be expected if these animals have the incisor genetic module identified by previous studies on mouse and primate quantitative genetics (Hlusko et al. 2011; Hardin 2020). This pattern is most evident in the mandible of *Canis*, the maxilla and mandible of *Colobus*, and to a lesser degree in *Didelphis, Ursus,* and *Odocoileus*. While the evidence for separate incisor and post-canine modules in our analyses is not as clear as that reported in previous genetic analyses of baboons, mice, and macaques (Hlusko et al. 2011; Hardin 2020), we interpret our phenotypic results as tentative evidence of underlying genetic modularity that differentiate the incisors from the post-canine teeth. If true, this genetic architecture may have facilitated the evolution of the diverse forms of mammalian incisors observed today (e.g., elephant tusks, ever-growing incisors of rodents, the specialized incisor morphology of aye-ayes and lemurs, and the loss of maxillary incisors in cervids; Hlusko et al. 2011).

In contrast, our phenotypic analyses do not provide support for the interpretation of molar: premolar genetic submodularity across mammals other than in our sample of the primate *Colobus*, rejecting our second hypothesis that the premolar:molar modularity is ancestral to eutherians and perhaps even to metatherians. Unlike for the incisors, where they were generally in the same cluster whether we estimated three, four, or five clusters in the hierarchical clustering analysis, the post-canine teeth vary considerably in their clustering pattern across taxa. For example, while the *Odocoileus* hierarchical-clustering analysis consistently groups the two mesial molars together, the most-distal third molar clusters either with the premolars or by itself, for both the maxilla and mandible. In the hierarchical clustering for the maxillary dentition of *Didelphis*, a marsupial, the only consistent clustering in the post-canine dentition is among P3, M3, and M4. The remaining teeth (I1, I5, P1, P2, M1, M2, and M5) do not cluster by tooth category. The *Didelphis* mandibular post-canine dentition does not cluster by tooth type.

The rejection of the hypothesis of premolar:molar submodularity in the post-canine dentition of mammals generally is, perhaps, not surprising. Genetic modularity, especially of the posterior dentition, appears to be responsive to selective pressures. For example, an investigation of morphological modularity in secondarily-derived homodont pinnipeds found no evidence of posterior modularity (Wolsan et al. 2019). However, the level of integration across the pinniped dentition is higher than what has been reported for mammals with more complex dentitions (Wolsan et al. 2019). Placing this into context with the higher degree of integration and lack of modularity similarly observed for tamarins (Hardin 2019), perhaps post-canine modularity is readily responsive to selective pressures over evolutionary time, dissipating and reformulating in different patterns. The results from our analysis of two carnivores support this idea.

The two carnivore taxa in our study yield variational patterns in the post-canine dentition that center around the carnassial teeth. Our hierarchical clustering analysis groups the *U. americanus* maxillary carnassial (P4) with molars, and the *C. latrans* mandibular carnassial (m1) with premolars. Additionally, in the *C. latrans* maxilla, we observe the centering of the variational module around the carnassial tooth (P4). It is interesting that these two carnivores in our study, who have different diets and carnassial morphology, both exhibit grouping with respect to the carnassial teeth. In carnivorans, the carnassial teeth (maxillary P4 and mandibular m1) form a shearing complex adapted to provide a specific maximal bite force in the dentition, and the morphology of carnassial teeth among carnivore taxa varies according to diet (Greaves 1983; Biknevicius and Van Valkenburgh 1996; Van Valkenburgh 1996). The carnassial teeth of the omnivorous *U. americanus*, for example, have lost the shearing function and instead more closely resemble the grinding surfaces of the post-carnassial molars (Sacco and Van Valkenburgh 2004; see Fig. 1). Our results perhaps reflect the unique selective pressures that have operated on the carnassial complex, which in turn affected the phenotypic variation of the entire post-canine dentition (for more discussion on carnassial genetics and evolution, see Van Valkenburgh 1991, 2007; Meloro and Raia 2010; Asahara 2013; Asahara et al. 2016; Tarquini et al. 2020). We present this hypothesis as one possible interpretation of the data, as we do see instances in non-carnivoran species where a certain tooth groups with teeth outside of their tooth class.

Of the taxa included in this study, we observe an unusual difference in the correlation estimates of male *O. hemionus* compared to females. Qualitatively, males of this species have higher degrees of inter-tooth correlation than do females. This sex difference was surprising given that there is no obviously discernable difference between female and male *O. hemionus* dental morphology. However, *O. hemionus* was the only species in our study with a notable sexually dimorphic cranial feature: the presence of antlers in the male, but not female, skull (Anderson and Wallmo 1984).

Antlers, a headgear composed of exposed bone that is unique to cervids, shed and regenerate annually (Davis et al. 2011). The life cycle of the antler is closely tied to seasonally fluctuating androgen levels (reviewed in Price et al. 2005; Davis et al. 2011; Kierdorf and Kierdorf 2011; Li et al. 2014). In male *Odocoileus*, antler development starts in the first year of life (French et al. 1956; Davis et al. 2011). The permanent incisors of *O. hemionus* develop between 12 and 18 months of age, and the permanent premolars develop between 24 and 29 months of age (Robinette et al. 1957). Molars, which are the only tooth class that do not undergo replacement in eutherians (Luo et al. 2004), start eruption at 3 months and complete growth by 28 months (Robinette et al. 1957). The overlapping timeline in the development of permanent dentition and antler development and shedding presents the intriguing, though tentative, hypothesis that androgens responsible for the antler life cycle may have a secondary, non-adaptive effect on the tooth size variation of male *Odocoileus*. This is bolstered by the observation that steroid receptors in dental pulp cells are implicated in dental and enamel development in rats and humans (Pirinen 1995; Dale et al. 2002; Inaba et al. 2013; Jedeon et al. 2016). Furthermore, there is evidence of steroids affecting the skeletal proportion of mammals: the sex difference of digit ratios in mice results from the intersexual differences in androgen and estrogen signaling during embryonic development (Zheng and Cohn 2011).

There are several limitations to this study. The data were collected from specimens from wild, unpedigreed populations, which precludes direct testing of whether the observed phenotypic correlations result from underlying genetic correlations or from non-heritable components. A related complicating factor is the difficulty in knowing whether an insignificant phenotypic correlation results from (1) a lack of genetic correlation, (2) a net balance of genetic and environmental factors that counteract each other, or (3) low statistical power. Teeth missing from museum specimens (e.g., *U. americanus* premolars) or species with relatively smaller sample sizes (e.g., *D. virginiana*) may have reduced our ability to detect phenotypic correlations. *U. americanus* also had specimens of unknown sex, which may have diluted the power of sex-difference analyses for this species. Finally, with the exception of the previously collected *C. guereza* dataset, we were logistically limited to sampling North American species that were available in large numbers at University of California's Museum of Vertebrate Zoology. Despite these limitations, we identified conserved and species-specific patterns of phenotypic variation in the dental arcades of four eutherian and one marsupial species. Furthermore, the characterization of phenotypic dental modularity in non-traditional models provides unique and useful perspectives that can serve as starting points to generate hypotheses for future studies.

## Supplementary Material

obac017_Supplemental_Files
