# Supplementary material for: Conserved and Taxon-Specific Patterns of Phenotypic Modularity in the Mammalian Dentition
Source: Integr Org Biol. 2022 Apr 28;4(1):obac017. doi: 10.1093/iob/obac017 (PMC9191923; doi:10.1093/iob/obac017)
Supplement: obac017_Supplemental_Files [file obac017_supplemental_files.zip › SUPPLEMENTARY_FIGs_1,3,4,5_updated.docx]

**
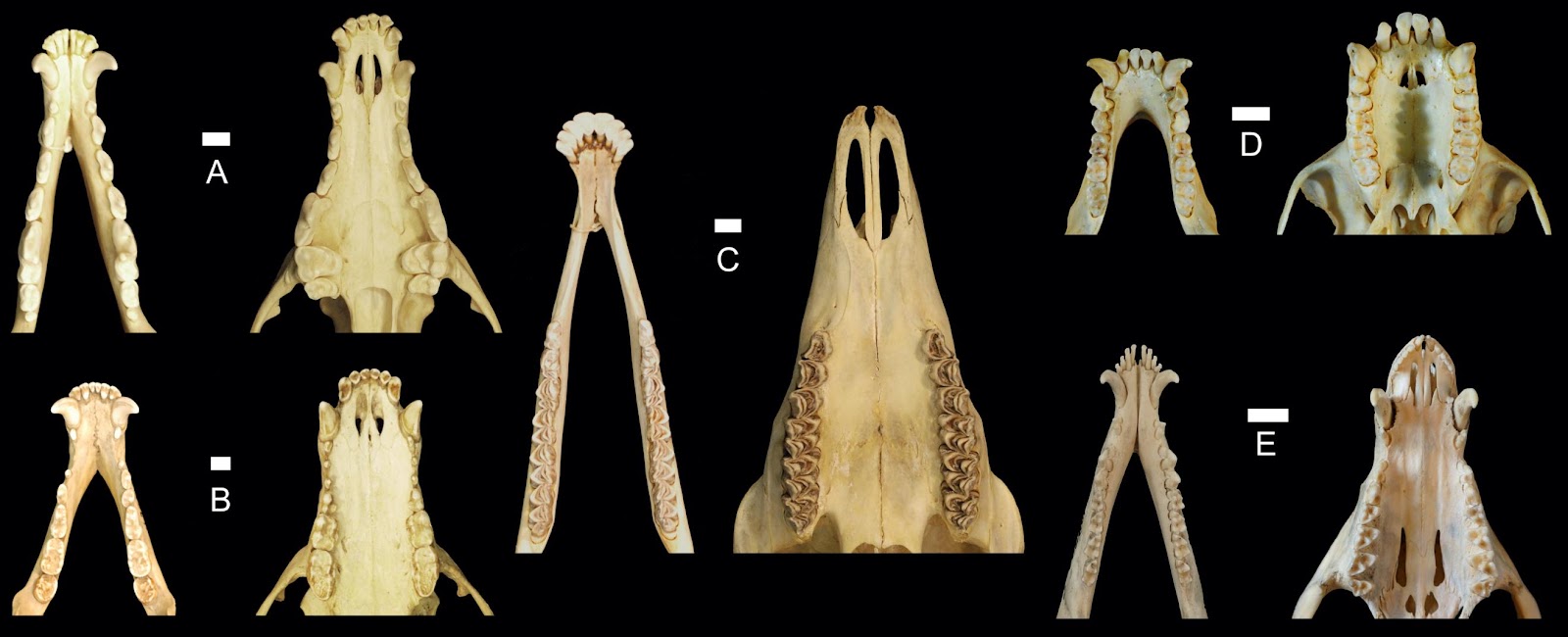
**

**Supplementary Figure 1**. **Representative mandibular (left) and maxillary (right) dentitions of the five taxa included in this study.** All are shown in occlusal view with the labial surface oriented to the top of the figure. All specimens are sized so that the breadth across the maxillary arches is approximately equivalent, to give the reader a sense of the relative size variation along the dental arcades rather than the relative sizes of the various species. The white bars above each of the identification letters represents 1cm for that specimen. A) *Canis latrans*, B) *Ursus americanus,* C) *Odocoileus hemionus*, D) *Colobus guereza*, E) *Didelphis virginiana*.

**Supplementary Figure 2**. **Phenotypic correlation matrices with length and width measurements.** See separate Excel file.


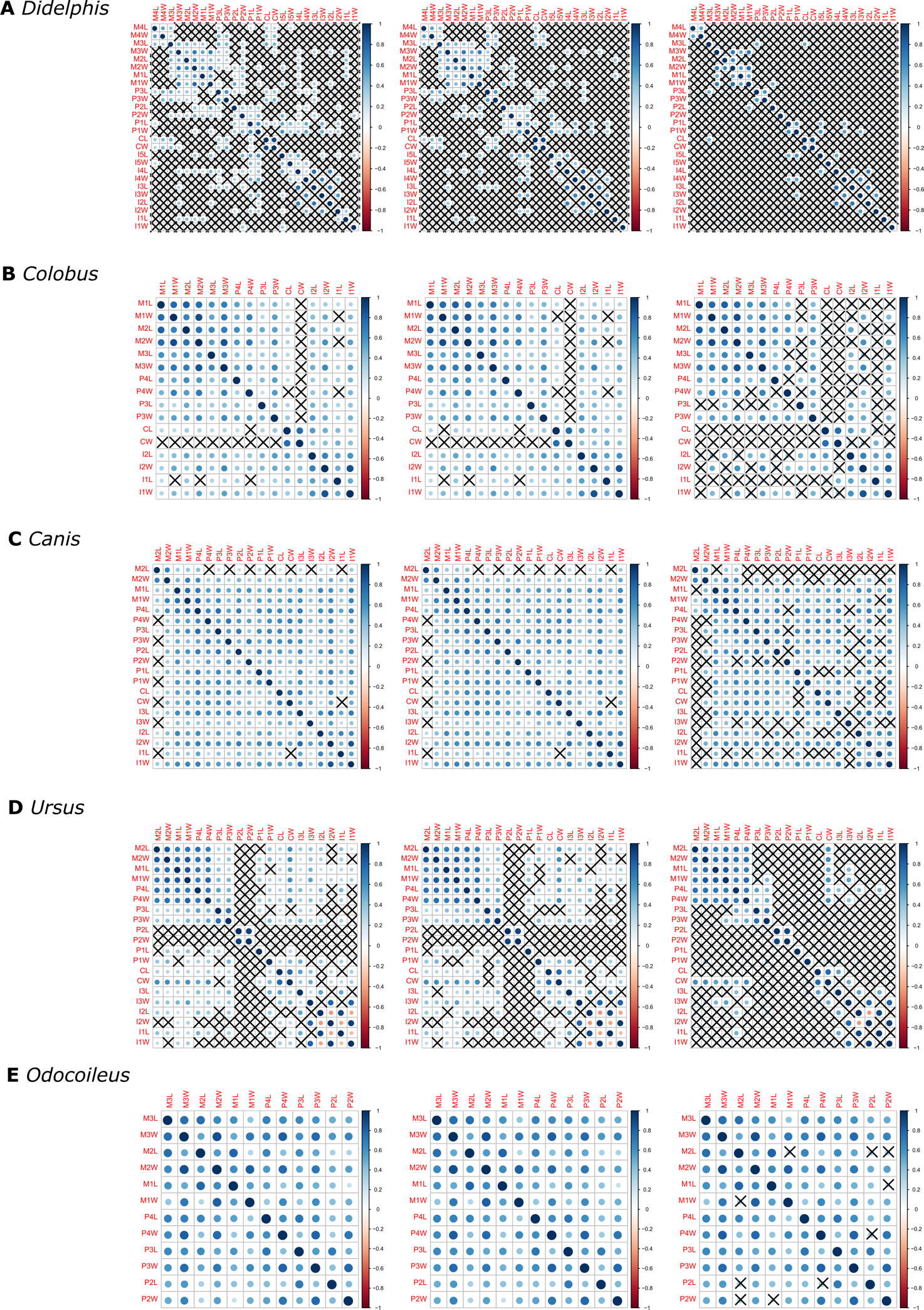

**Supplementary Figure 3. Correlation matrices with p-value corrections for the maxilla.** The strength of the correlation is denoted by color and dot size, with stronger correlations in darker and larger dots. Blue and red dots indicate positive and negative correlation, respectively. An X over the cell indicates the correlation is not significant. Three plots are shown for each species: the left plot shows the results where the raw (uncorrected) p-value is not significant (threshold of 0.05), the middle plot shows the results of using an FDR of 0.05 threshold, and the plot on the right shows the results from applying a Bonferroni correction. Matrices are symmetrical across the diagonal.


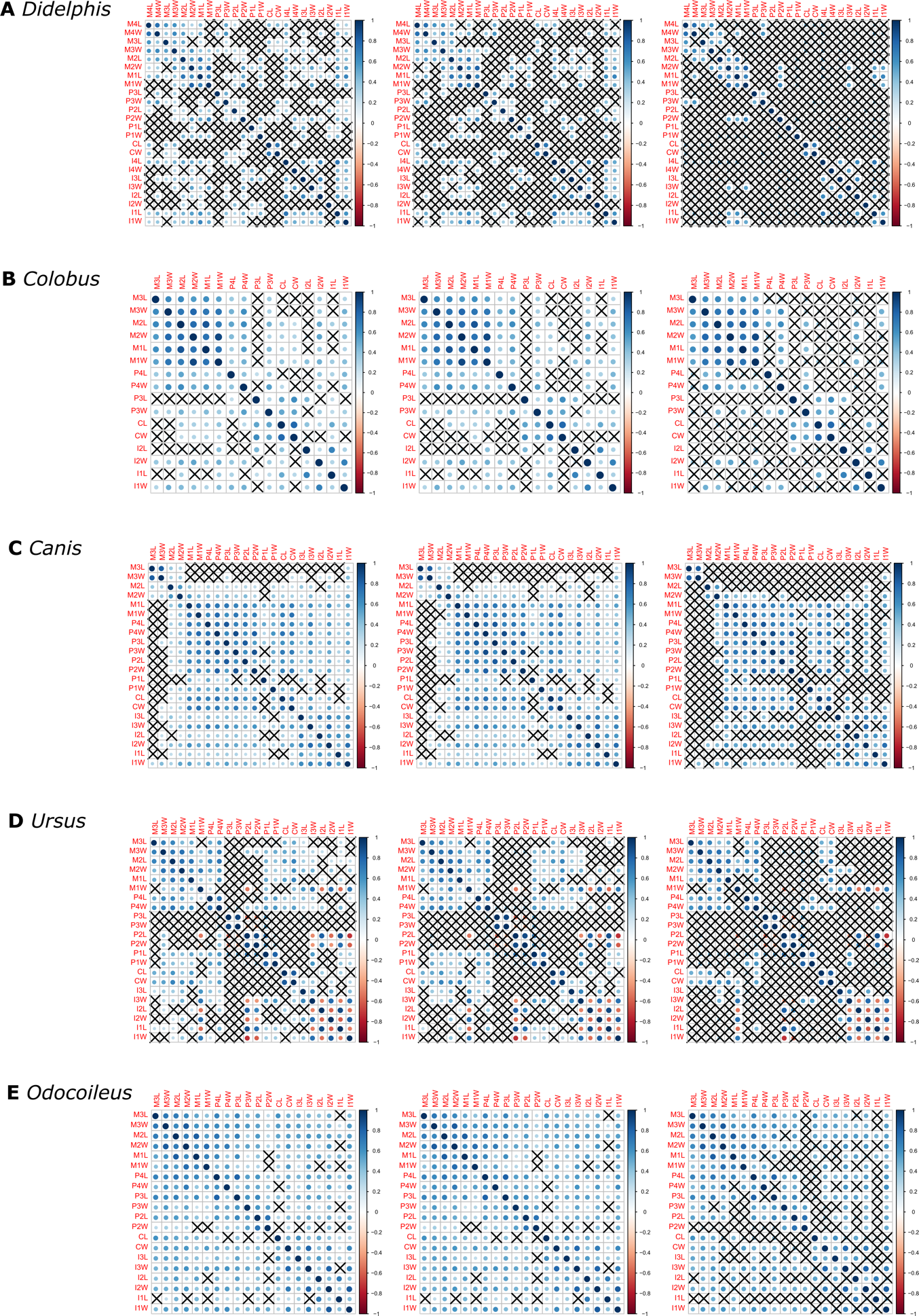

**Supplementary Figure 4.** **Correlation matrices with p-value corrections for the mandible.** The strength of the correlation is denoted by color and dot size, with stronger correlations in darker and larger dots. Blue and red dots indicate positive and negative correlation, respectively. An X over the cell indicates the correlation is not significant. Three plots are shown for each species: the left plot shows the results where the raw (uncorrected) p-value is not significant (threshold of 0.05), the middle plot shows the results of using an FDR of 0.05 threshold, and the plot on the right shows the results from applying a Bonferroni correction. Matrices are symmetrical across the diagonal.


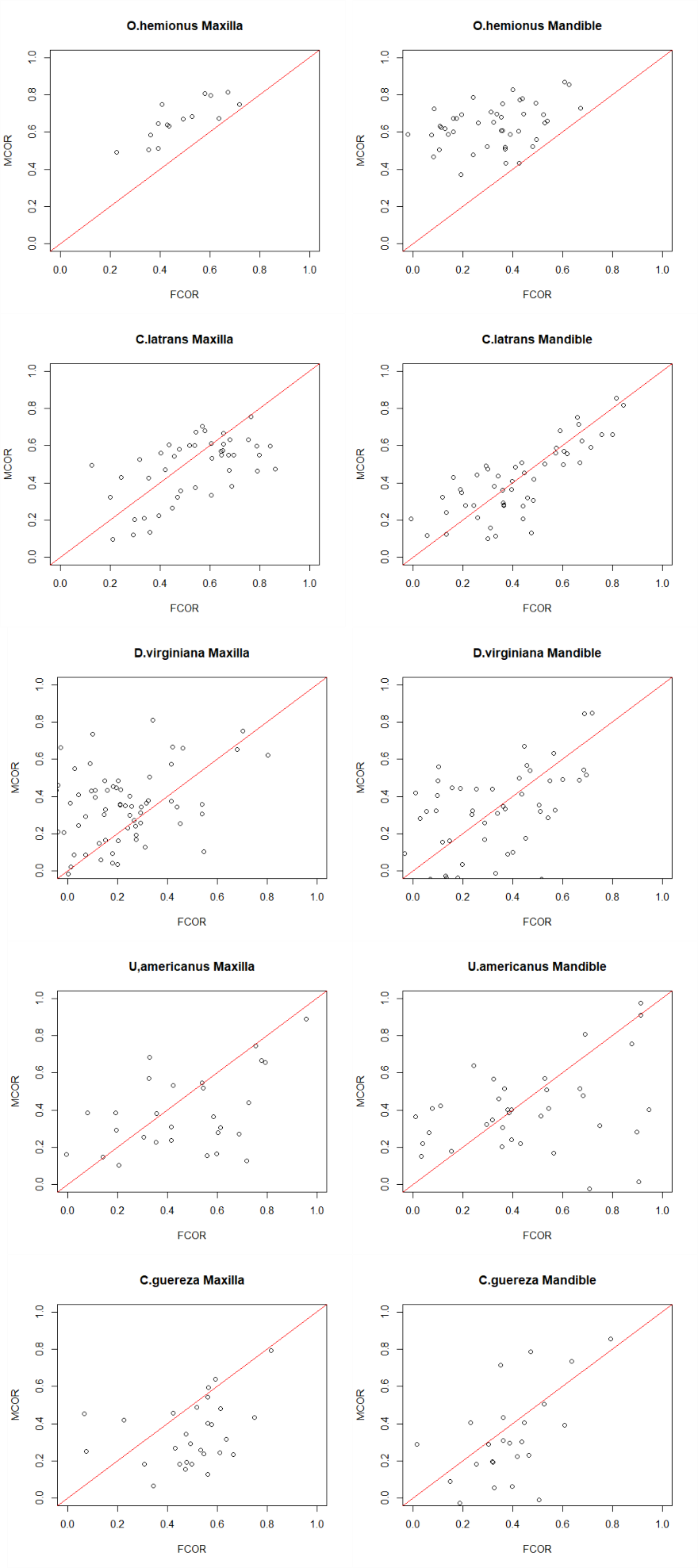


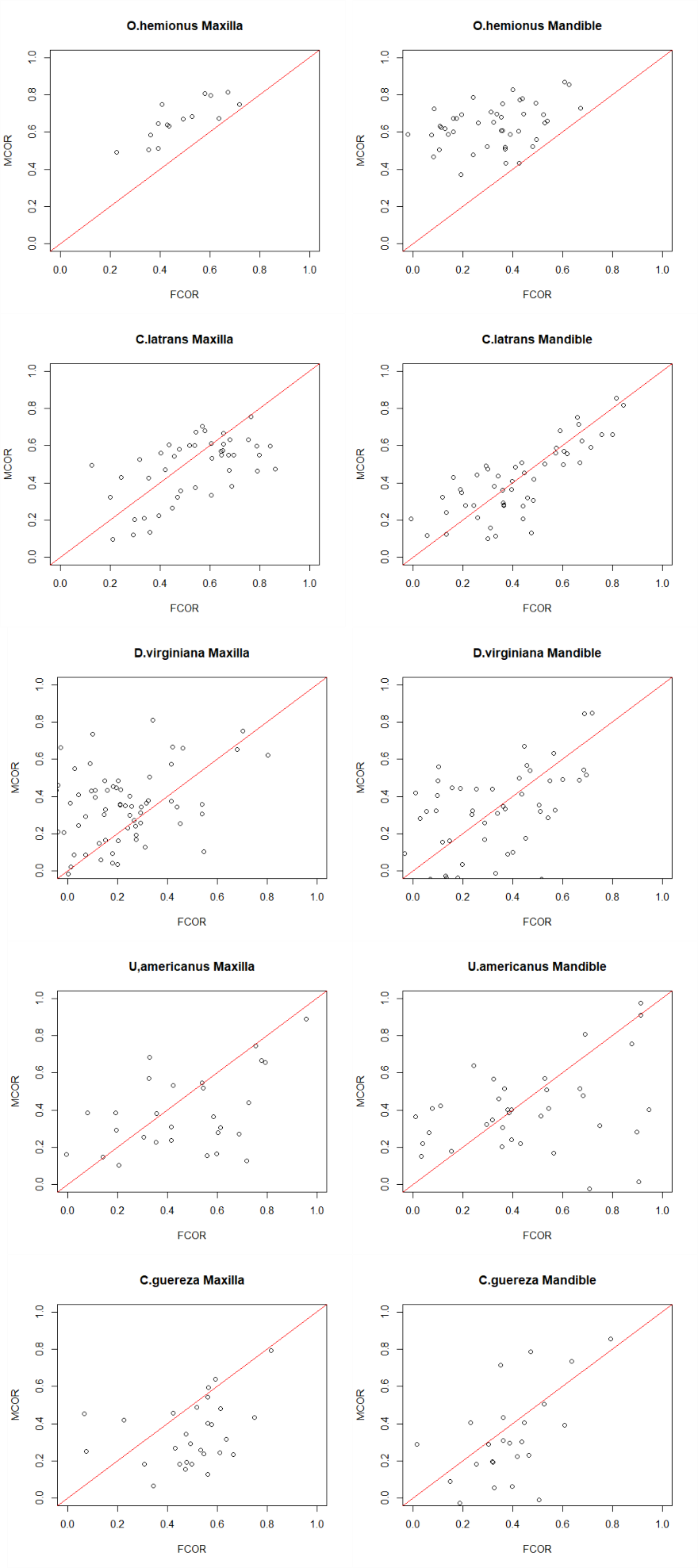


**Supplementary Figure 5.** **Correlation matrices with p-value corrections for the mandible.** Scatterplots show male vs female correlation values for each pair of teeth. Only length measurements were used, and results were plotted for the maxilla and mandible of each species. MCOR and FCOR correspond to correlation values from the male and female specimens, respectively. Of these, *O. hemionus* maxilla and mandible show that males have overall higher correlations than the females do for the corresponding teeth.
